# Early maternal age at first birth is associated with chronic diseases and poor physical performance in older age: cross-sectional analysis from the International Mobility in Aging Study

**DOI:** 10.1186/1471-2458-14-293

**Published:** 2014-03-31

**Authors:** Catherine M Pirkle, Ana Carolina Patrício de Albuquerque Sousa, Beatriz Alvarado, Maria-Victoria Zunzunegui

**Affiliations:** 1 Department of Population Health and Environment, Research Center CHUQ, Laval University, 2875, Boulevard Laurier, Édifice Delta II; Bureau 600, 6e étage, Québec, QC G1V 2M2, Canada; 2 Department of Physiotherapy, Universidade Federal do Rio Grande do Norte, Avenida Senador Salgado Filho, S/N Caixa Postal 1524 - Campus Universitário - Lagoa Nova CEP, Natal, RN 59072-970, Brazil; 3 Department of Public Health Science, Queen’s University, Carruthers Hall, Queen's University Kingston, Kingston, ON K7L 3 N6, Canada; 4 Centre de Recherche du Centre Hospitalière de l’Université de Montréal, Institute de Recherche en Santé Publique de l’Université de Montréal, Tour Saint-Antoine, 850, rue St-Denis, Suite S03.312, Montreal H2X 0A9, Québec, Canada

**Keywords:** Global health, Life-course epidemiology, Aging, Adolescent pregnancy, Parity, SPPB

## Abstract

**Background:**

Early maternal age at first birth and elevated parity may have long-term consequences for the health of women as they age. Both are known risk factors for obstetrical complications with lifelong associated morbidities. They may also be related to diabetes and cardiovascular disease development.

**Methods:**

We examine the relationship between early maternal age at first birth, defined as ≤18 years of age, multiparity (>2 births), and poor physical performance (Short Physical Performance Battery ≤8) in community samples of women between 65 and 74 years of age from Canada, Albania, Colombia, and Brazil (N = 1040). Data were collected in 2012 to provide a baseline assessment for a longitudinal cohort called the International Mobility in Aging Study. We used logistic regression and general linear models to analyse the data.

**Results:**

Early maternal age at first birth is significantly associated with diabetes, chronic lung disease, high blood pressure, and poor physical performance in women at older ages. Parity was not independently associated with chronic conditions and physical performance in older age. After adjustment for study site, age, education, childhood economic adversity and lifetime births, women who gave birth at a young age had 1.75 (95% CI: 1.17 – 2.64) the odds of poor SPPB compared to women who gave birth > 18 years of age. Adjustment for chronic diseases attenuated the association between early first birth and physical performance. Results were weaker in Colombia and Brazil, than Canada and Albania.

**Conclusions:**

This study provides evidence that adolescent childbirth may increase the risk of developing chronic diseases and physical limitations in older age. Results likely reflect both the biological and social consequences of early childbearing and if the observed relationship is causal, it reinforces the importance of providing contraception and sex education to young women, as the consequences of early pregnancy may be life-long.

## Background

As populations age, physical decline, which includes loss of muscle strength, balance, and mobility, as well as decreased gait speed, constitutes an important public health concern. It is a major step in the disablement process and consequences of physical decline include diminished quality of life, increased healthcare expenditures, and death [1,2]. Worldwide, it has been documented that as populations age, women experience worse physical function and greater physical decline than men at similar ages [1,3-8]. Increasingly, there is evidence that the physical function differences between women and men are heterogeneous across settings and these are linked to gender inequality. Countries with the greatest gender inequalities also have the highest odds-ratios for mobility disability in women compared to men [4]. At the individual level, the disability gap between women and men is even more substantial among people with low education and low income [5,9-11].

A number of biological and social explanations are proposed to explain women’s relatively greater burden of physical decline and disability. On the one hand, it may arise from a higher cumulative burden of physiological dysregulation, especially in the postmenopausal period [12,13], as well as a greater risk of diseases (arthritis, osteoporosis and depression) that hamper physical function [6]. On the other hand, the greater burden has also been linked to gender inequality accumulated throughout the life-course [4]. Gendered norms, values and behaviours contributing to differences in educational attainment, physical activity, smoking, diet, childhood hunger, poverty, and body mass index may all predispose women to physical decline and disability [8,14]. However, research on the roles of these factors has been fragmentary and can only explain a fraction of the sex/gender gap [1,8,15]. In contrast, relatively little attention has been paid to the role of women’s reproductive history on physical decline in older age. Women in lower income settings tend to start childbearing earlier, have more children, and face more risks during childbirth [16] {Glasier, 2006 #94]}. Their reproductive histories may partially account for the greater prevalence and earlier onset of physical decline documented in these settings, especially if early childbirth and higher parity affect future life opportunities.

Pregnancy fundamentally alters a woman’s physiology and increases metabolic demands on the body [17]. When women begin giving birth at a young age and/or give birth to numerous children, permanent physiological alterations may occur that increase the risk of chronic diseases and poor physical functioning in older age. For example, pregnancy may interfere with bone mineral density and increase the future risk of osteoporosis, especially in women who give birth as adolescents [18]. Pregnancy may also alter long-term glucose homeostasis [19] and multiple studies have documented associations between multiparity and type-II diabetes in later life [19-21]. Both multiparity and early maternal age at first birth have been associated with diabetes-related death [20]. Parity has also been associated with cardiovascular disease- including coronary heart disease, stroke, and heart failure- in later life [22].

While there is a growing body of evidence supporting a link between reproductive history and chronic disease, very little research has investigated a link between reproductive history and physical function in older age. Childbearing, especially in women with limited food supplies, can drain nutritional reserves [17,23] that may protect against loss of mobility later in life. Similarly, numerous births and particularly complicated births can permanently damage the bones, ligaments, and nerves around the pelvis and hips, giving rise to gait abnormalities and drop foot (inability to dorsiflex the foot) [24]. Pelvic floor disorders (prolapse, incontinence, overactive bladder) are prevalent in elderly women in low and high-income settings and often follow multiple vaginal deliveries [24,25]. Severe pelvic floor disorders have implications for mobility and in the case of lost urinary/fecal control, the stigmatization associated with these conditions can fundamentally alter life opportunities [24] with indirect consequences for physical functioning.

In this paper, we assess whether there is a link between reproductive history and physical function in older age. Specifically, we examine the associations between young maternal age at first birth and multi-parity on chronic diseases and poor physical function in older age in community-dwelling elderly from five research sites (Quebec and Ontario in Canada, Albania, Colombia, and Brazil). We hypothesize that women who gave birth as teenagers and women who have had numerous children will have a greater prevalence of chronic diseases than women who gave birth older and had fewer children. We also hypothesize an independent association between early maternal age at first birth and elevated parity with poor physical function in older age. Finally, because women from the Latin American sites would have had less access to high quality obstetrical care during their childbearing years, we hypothesize that any relationship between early maternal age at first birth and parity with physical function will be more profound in Latin America than the Canadian and European sites.

## Methods

### Context

This research was conducted as part of a longitudinal study, called The International Mobility in Aging Study. The primary objective of IMIAS is to measure the magnitude of the sex/gender gap in mobility and to increase understanding of sex/gender differences in life-course exposures related to mobility. IMIAS is ongoing at five sites: Tirana (Albania), Natal (Brazil), Manizales (Colombia), Kingston (Ontario, Canada) and Saint-Hyacinthe (Quebec, Canada). These cities were chosen because each has a relatively homogenous elderly population in terms of life opportunities, religion, ethnic diversity and national origins. Across sites, they represent societies that vary considerably in gender equality. According to the United Nations 2012 Gender Inequality Index of 148 countries, Canada was 18th while Albania, Brazil, and Colombia were ranked 41st, 85th, and 88th, respectively [26]. This diversity across populations can be used to describe differences in prevalence that cannot be explained by individual risk factors [27] and to increase the range of exposures and the prevalence of health and functional outcomes. Below, we present contextual details for each site, highlighting local circumstances affecting childbearing at each setting.

Tirana, the capital of Albania, is a city of approximately 700,000 inhabitants. Albania is a post-communist republic and one of the poorest countries in Europe. During the 1960s, when most women in this cohort were giving birth, Albania experienced remarkable fertility decline, despite restricted access to birth control. Childbirth outside of marriage in Albania was exceedingly rare [28]. Natal, in northeast Brazil, has 800,000 inhabitants and is the capital of the province of Rio Grande do Norte, which is one of the most impoverished regions of Brazil. Historically, there have been significant barriers to quality obstetrical care in Brazil and maternal and child health indicators were poor [29]. Manizales has a population of 400,000 and is found in the relatively affluent coffee-growing zone of the Colombian Andes Mountains. When women in this cohort were giving birth, there was strong opposition to contraception by the Catholic church [30]. As in Brazil, maternal and child health indicators have been poor [31]. In Canada, Kingston is a city with 130,000 inhabitants located in Ontario. The population is English-speaking and mostly Christian. Finally, St. Hyacinthe has a population of about 50,000, about 50 Km from Montreal, Quebec. The community is predominantly Catholic and French-speaking.

### Study design

This is a cross-sectional analysis of baseline data from IMIAS.

### Population

The study is composed of community dwelling elderly people between 64 and 75 years of age. The sample was stratified by sex with an aim to recruit 200 men and 200 women from each site. The total sample size of women in the study is 1040.

### Sampling strategy

Baseline data were collected in 2012: from January to June in Manizales, Natal, and St Hyacinthe; from January to December in Kingston; and from September to December in Tirana. Follow-up data collection phases are planned for 2014 and 2016. In Tirana, Manizales and Natal, participants were recruited through neighbourhood primary care centers. At these sites, a random sample of elderly people registered at the health center was drawn and participants were approached directly by our interviewers to invite them to participate. Ethics’ committees at both Canadian Universities did not allow researchers to directly contact potential participants. Invitations to participate in the project were sent indirectly via family physicians. Potential participants received a letter from their family physician inviting them to contact our field coordinator for information about the study. Since Albania, Brazil and Canada have universal health care systems, more than 90% of the population aged 64 to 75 is registered at a health center or has a primary care physician. In Colombia, it is estimated that approximately 82% of individuals in this age group are registered in the public health system [31].

Response rates were greater than 90% in Tirana and close to 100% in Natal and Manizales. In Kingston and St Hyacinthe, only 30% of people receiving a letter from their doctor contacted us. However, of those who telephoned to obtain more information about the study, 95% agreed to participate, resulting in overall response rate of 28%. At both Canadian research sites, responses rates were lower among men than women.

### Exclusion criterion

Participants were excluded if they had 4 or more errors on the orientation scale of the Leganes Cognitive Test [32], which was administered at the beginning of the study interview. Low scores on the orientation scale were considered indicative of inability to complete the study procedures.

### Sample size

Given the principal objective of IMIAS, total sample size was calculated to estimate sex and site-specific prevalence, incidence, and recovery from mobility disability. Of relevance to the present study, we estimated the prevalence of mobility disability- a consequence of functional decline- to range between 16 and 27% in this age group. Thus, with 200 women at each site at baseline, we have the following level of precision for the prevalence of mobility disability: 27% ± 6%, n = 200. Prior to commencing IMIAS, sample size was also estimated to assess the sex-specific relationship between life-course disadvantage exposures, such as early maternal age at first birth, and chronic conditions. For a chronic condition like heart disease, we have 80% power to detect an odds ratio of 2, assuming a prevalence of heart disease of 10% and a prevalence of a life-course disadvantage of 20%.

### Data collection

At all sites, study procedures were carried out at the participant’s home unless that person requested otherwise. In Manizales, physical performance was evaluated at the local hospital.

Interviewers at each site received the same standard training based on videotapes, protocol instructions and data entry forms. Assessments in Tirana were done by public health professionals and graduate students, in Natal by physiotherapists, in Manizales by local nurses, in Saint Hyacinthe by retired nurses and teachers, and in Kingston by retired professionals with at least a high school education. The principal investigators and the study coordinator trained all interviewers. The questionnaires, data collection documents and procedures manuals were available in the local languages: Albanian, Spanish, Portuguese, English and French. They are accessible at our web site (http://www.imias.umontreal.ca). French, Portuguese and Spanish versions of the main scales were validated in two pilot studies conducted in Brazil, Colombia and Quebec [33].

### Reproductive history exposure measures

Age at first birth and parity were self-reported. Age at first birth was dichotomized as: first birth at 18 years of age or younger and first birth at 19 years of age or older. The cut-off of 18 years was selected based on evolutionary theories about an “optimal age of first birth” in humans [34,35]. Accordingly, optimal age at first birth is a trade-off between accumulating sufficient nutritional reserves for pregnancy, maximizing fertility while reducing risk of obstetrical complication, and achieving maximal successful reproduction [34]. The role of parity was assessed both as a continuous and dichotomous variable (2 or fewer children and 3 or more children). The cut-off of two or fewer children was selected based on evidence that 3 or more children is associated with coronary heart disease, stroke, and heart failure [22].

### Covariates

Age was centred at 64 (reported age minus 64). Sixty-four was the age at which a woman was eligible to participate in the study. Education was dichotomized as: 8 years of schooling or less and 9 or more years of education. As girls may drop out of school as a result of pregnancy, we chose to dichotomize education at an age at which most women would not become pregnant (13/14 years of age). In our sample, only eight women had children at 14 years of age or younger. Childhood economic adversity was measured with a scale varying from 0 to 3. A point was given for each of the following self-reported childhood circumstances: poverty, hunger, and parental unemployment. We did not adjust for adulthood measures of socio-economic status, such as income and occupation, because professional opportunities are affected by reproductive history and controlling for these attributes would lead to over-adjustment [36].

Early maternal age at first birth and multiparity may be associated with chronic diseases that, in turn, may be associated with poor physical performance. Chronic diseases- osteoporosis, diabetes, chronic lung disease, high blood pressure, stroke, coronary heart disease, arthritis, and cancer- were self reported based on the question “Has a doctor or nurse ever told you that you have …”.

### Outcome measure: physical performance

Physical performance was assessed by the Short Physical Performance Battery (SPPB) [37,38]. The SPPB includes three tests of lower body function: a hierarchical test of standing balance, a 4-meter walk, and five repetitive chair stands. Each SPPB component test (balance, gait and chair stand) is scored from 0 to 4 with a score of 0 representing inability to perform the test and a score of 4 representing the highest category of performance. Scores can vary between 0 and 12 with greater scores measuring better physical performance. Poor physical performance was defined as 8 or below. Scoring cut-off points were derived from a large representative population of older persons. The SPPB has been validated for use in Spanish and Portuguese in Colombia and Brazil, respectively [33].

### Statistical analyses

Statistical analyses were conducted using STATA/SE 13. Student’s *t*-test and two-by-two tables were employed to describe exposure measures, covariates, and poor physical performance. Because chronic diseases were believed to be intermediate variables on the pathway between reproductive exposures and physical performance, we first used logistic regression to assess associations between exposure variables- age at first birth and parity- and reported chronic conditions, with adjustment for study site. Next, for the dichotomous SPPB outcome variable, we used three logistic regression models. In the first model, our independent variables were maternal age at first birth and the potential covariates: centred age variable, education, childhood economic adversity and study site. In our second model, we added parity as a possible intermediate variable and in the third model, we added chronic diseases as a possible pathway for the effects of age at first birth on physical function. Age at first birth and parity were not assessed simultaneously because parity may be a function of the age at first birth (e.g. the younger a woman begins childbearing, the more opportunity she has for more children). An identical modeling strategy was used for the continuous SPPB outcome. Finally, using the continuous SPPB outcome, we assessed for multiplicative interaction between study site and age at first birth.

### Sensitivity analyses

In the multivariate models with parity, we assessed different cut-off values for the variable. These included: 1) 0 births, 1–2 birth, 3 or more births; 2) 0 births, 1–2 births, 3–4 births, 5 or more births; 3) 0–4 births or 5 or more births.

### Analytic model

**Figure 1:**
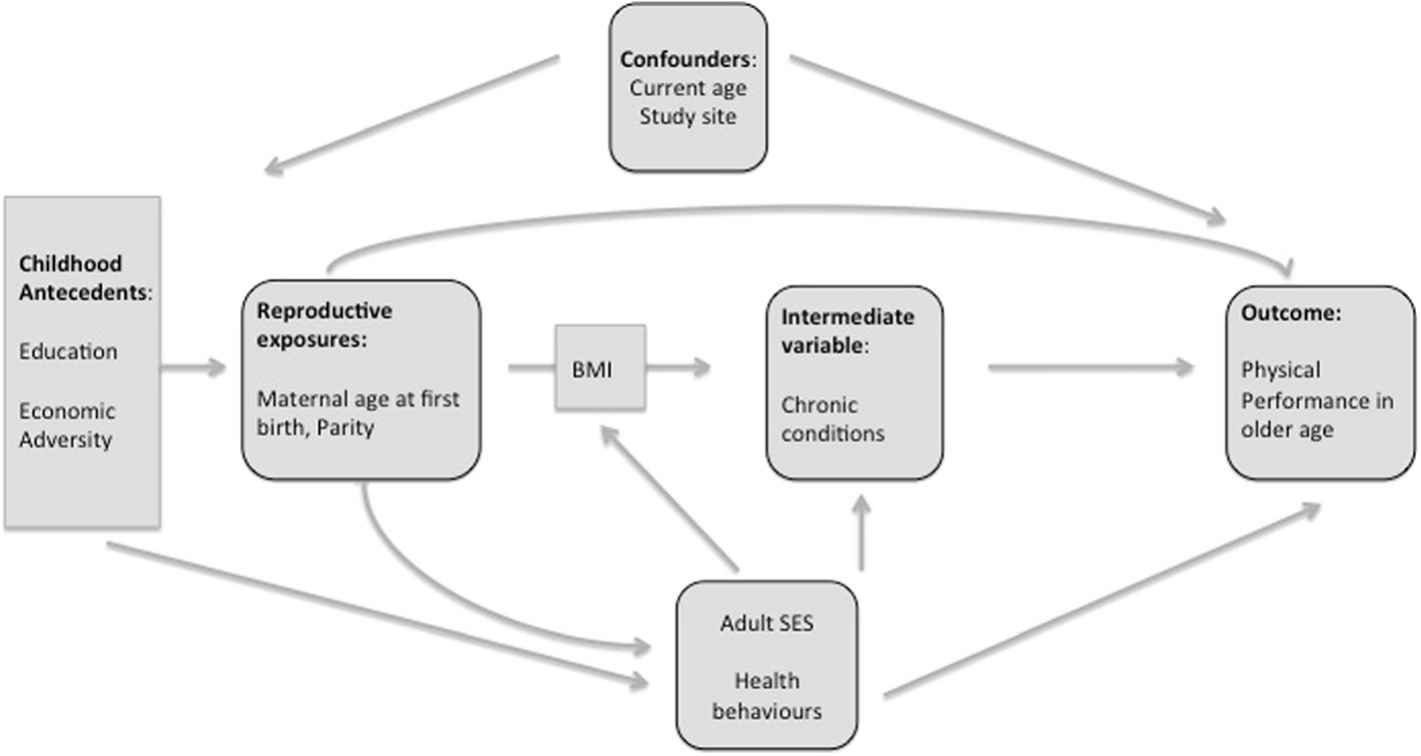
Analytic model of assumptions about the causal relationship between variables.

Figure 1 depicts our assumptions about the causal relationship between variables. It should be noted that since we hypothesize variables such as adult socioeconomic status, health behaviours, and BMI are on the causal path between our exposure and outcome variables, their inclusion in the statistical models would lead to over-adjustment.

### Ethics

Ethical approval for this project was obtained from the Research Centres of the University of Montreal Hospital Complex (CR-CHUM), the Albanian Institute of Public Health, and the research ethics boards of the Federal University of Rio Grande do Norte (Brazil), Queen’s University (Kingston) and University of Caldas (Columbia). All study participants provided written informed consent. In Brazil and Colombia, some participants could not read and write. The consent form was read to them, oral consent was obtained, and the participant placed a sign on the form to acknowledge agreement.

## Results

Fertility history in Kingston and St. Hyacinthe was similar; few women gave birth at less than 18 years of age and most women had their first child at 24 or 25 years of age. In the Latin American countries, the average age of first birth was 21 or 22 and a third of women had their first child at 18 years of age or younger. In Albania, women had children a younger mean age than in Canada, but very few gave birth as adolescents. Parity was also markedly higher at the Latin American sites with larger variation in the number births by these women, when compared to women from Canada and Albania. The majority of women in Latin America gave birth 3 or more times (Table 1). Finally, having a child at 18 years of age or younger was strongly associated with parity (p < 0.00). Only 12 (8%) women who gave birth at an early maternal age had 2 or fewer children compared to 359 (48%) women who had children at 19 years of age or older. Further, 64% of women who started childbearing at a young age gave birth 5 or more times (only 19% of women who gave birth later had 5 or more children).

**Table 1:** Age at first birth and parity by study site (N = 1040)

	**Kingston**	**St Hyacinthe**	**Tirana**	**Manizales**	**Natal**
	**(*n*** **= 209)**	**(*n*** **= 206)**	**(*n*** **= 204)**	**(*n*** **= 200)**	**(*n*** **= 210)**
Age at 1st birth^1^, mean (SD), yr	25.1 (5.2)	24.3 (4.1)	23.1 (4.1)	21.6 (5.4)	22.2 (5.7)
Women ≤ 18 yr at 1st birth, *n* (%)	17 (9.3%)	11 (6.4%)	16 (8.4%)	60 (33.3%)	55 (29.0%)
Parity, mean (SD)	2.1 (1.4)	2.1 (1.3)	2.8 (2.2)	4.9 (3.4)	4.9 (3.4)
Women with parity >2, *n* (%)	65 (31.1%)	74 (35.9%)	101 (49.5%)	153 (76.5%)	154 (73.3%)

^1^112 women never gave birth; missing age at first birth data for 14 women.

Educational attainment differed dramatically by site. Nearly all women at the Latin American sites had 8 or fewer years of education, while that was the case for half of the women in Tirana and for a quarter of women in St. Hyacinthe. Only 3% of women in Kingston had low educational attainment. Childhood economic adversity was greatest in Natal, similar in Tirana and Manizales, and lowest in Kingston and Quebec.

The prevalence of poor physical function was lowest in Canada (15%), followed by Manizales (26%), Albania (40%) and Natal (44%). The mean age of women in the sample was essentially the same at each site- 69 years (Table 2).

**Table 2:** Frequency measures for covariates by study site

	**Kingston**	**St Hyacinthe**	**Tirana**	**Manizales**	**Natal**
	**(*n*** **= 212)**	**(*n*** **= 210)**	**(*n*** **= 206)**	**(*n*** **= 202)**	**(*n*** **= 210)**
***Socio-demographic characteristics***					
Age, mean (SD), yr	69.1 (2.6)	68.6 (2.6)	69.2 (3.1)	69.3 (3.0)	69.3 (2.7)
≤8 yr education, *n* (%)	4 (2.6%)	45 (21.4%)	97 (47.1%)	172 (85.2%)	190 (90.5%)
Childhood economic adversity, mean (SD)^1^	1.0 (0.8)	1.3 (0.7)	1.6 (1.0)	1.5 (0.8)	2.0 (0.9)
***Chronic Conditions***^***2***^					
Diabetes, *n* (%)	23 (10.9%)	34 (16.3%)	60 (29.4%)	33 (16.3%)	66 (31.4%)
Stroke, *n* (%)	11 (5.2%)	11 (5.2%)	7 (3.4%)	12 (5.9%)	17 (8.1%)
Arthritis, *n* (%)	129 (60.9%)	116 (55.2%)	135 (65.5%)	79 (39.1%)	106 (50.5%)
Cancer, *n* (%)	36 (17.0%)	31 (14.8%)	5 (2.4%)	7 (3.5%)	8 (3.8%)
Chronic lung disease, *n* (%)	30 (14.2%)	26 (12.4%)	31 (15.1%)	34 (16.8%)	18 (8.6%)
Coronary heart disease, *n* (%)	34 (16.0%)	35 (16. 7%)	59 (28.6%)	33 (16.3%)	36 (17.1%)
Osteoporosis, *n* (%)	50 (24.1%)	52 (24.8%)	83 (40.3%)	45 (22.3%)	102 (48.6%)
High blood pressure, *n* (%)	89 (42.0%)	98 (46.7%)	152 (73.8%)	124 (61.4%)	155 (73.8%)
***Outcome***^***3***^					
SPPB, mean (SD)	10.2 (2.0)	10.0 (1.7)	8.4 (3.0)	9.4 (1.9)	8.4 (2.4)
SPPB ≤ 8, *n* (%)^4^	31 (14.6%)	32 (15.2%)	83 (40.3%)	50 (25.9%)	93 (44.3%)

^1^Childhood economic adversity scores can vary from 0 to 3. A point was given for each of the following self-reported childhood circumstances: poverty, hunger, and parental unemployment.

^2^Chronic diseases are self-reported measures.

^3^SPPB is the Short Physical Performance Battery; scores can vary from 0–12. Scores of 8 or below signify poor physical functioning.

^4^Missing 9 values for Manizales.

For each chronic disease, the proportion of individuals reporting diagnoses with the condition was similar at the two Canadian sites, although there was slightly more diabetes and slightly less arthritis in St. Hyacinthe. Tirana, Manizales, and Natal had much higher proportions (12 – 32% greater) of the population diagnosed with high blood pressure, compared to the Canadian sites. In contrast, these sites had much lower proportions diagnosed with cancer (11–15% less). In Tirana, compared to the Canadian sites, all chronic conditions were more prevalent, except for cancer and stroke. In Manizales, similar proportions of the population were diagnosed with most of the chronic conditions, compared to the Canadian sites. However, fewer reported having been diagnosed with arthritis and cancer and more reported high blood pressure. Compared to the Canadian sites, Natal had less reported arthritis, cancer, and chronic lung disease, but there was more osteoporosis and high blood pressure. Not one of the chronic diseases was similarly prevalent across all five sites (Table 2).

There was a significant direct association between early maternal age at first birth and a number of chronic disease outcomes: high blood pressure, diabetes, and chronic lung disease (Table 3). These associations remained highly significant, even after adjustment for age, education, and childhood economic adversity. For stroke, the association was marginal. However, after adjustment for age, education, and childhood economic adversity, the relation of early maternal age at first birth to stroke became less significant, although the odds ratio was only slightly smaller (OR = 1.75, 95% CI = 0.89 – 3.43, p-value 0.10). There was no significant association between early maternal age at first birth and osteoporosis, arthritis, cancer, or coronary heart disease (Table 3).

**Table 3:** **Association between age at first birth and parity and diagnosis with a chronic disease**^**1**^**, adjusted for study site**

	**≤ 18 yr at 1st birth**		**≥ 3 lifetime births**	
	**(N = 914) **^**2**^		**(N = 1029)**	
	**OR (95% CI)**	**p-value**	**OR (95% CI)**	**p-value**
High blood pressure	2.09 (1.38 – 3.16)	<0.01	1.64 (1.24 – 2.16)	<0.01
Diabetes	1.88 (1.25 – 2.83)	<0.01	1.71 (1.22 – 2.40)	<0.01
Chronic lung disease	2.40 (1.49 – 3.86)	<0.01	1.38 (0.93 – 2.04)	0.11
Stroke	1.91 (0.98 – 3.73)	0.06	1.25 (0.69 – 2.25)	0.46
Coronary heart disease	1.35 (0.87 – 2.09)	0.18	1.34 (0.95 – 1.87)	0.09
Osteoporosis	1.35 (0.92 – 1.99)	0.13	0.95 (0.71 – 1.26)	0.71
Arthritis	1.04 (0.72 – 1.51)	0.81	0.94 (0.72 – 1.23)	0.65
Cancer	1.10 (0.51– 2.38)	0.81	0.80 (0.49 – 1.30)	0.36

^1^Chronic diseases are self-reported measures.

^2^112 women never gave birth; missing age at first birth data for 14 women.

Having 3 or more children was significantly associated with diabetes and high blood pressure. This remained the case even after adjustment for age, education, and childhood economic adversity. Having three or more children was marginally associated with coronary heart disease, but the association was attenuated after adjustment for age, education, and childhood economic adversity (OR = 1.19, 95% CI = 0.84 – 1.70, p-value 0.31). Because the majority of women who gave birth at 18 years of age or less had 3 or more children, we further adjusted for age at first birth. The odds ratio for diabetes was 1.34 (95% CI = 0.91 – 1.96, p-value 0.14) and the odds ratio for high blood pressure was 1.39 (95% CI 1.01 – 1.91, p-value 0.04). In both models, age at first birth was highly significant (p = 0.01).

In the sensitivity analyses, we assessed whether different cut-off values for parity affected the observed associations. Compared to women who never gave birth, women who gave birth five or more times had 2.63 the odds of diabetes (p = 0.01), 2.10 the odds of chronic heart disease (p = 0.03), and 1.76 the odds of high blood pressure (p = 0.03). The magnitude of the association between parity and these chronic diseases was stronger than when we dichotomized the variable as three or more births versus two or fewer births. However, once adjustment was made for maternal age at first birth, the associations were no longer significant. Other cut-off values for parity, and modeling parity as a continuous variable, did not provide any additional information.

In models 1 – 2 of Table 4, age at first birth was significantly associated with poor physical performance in older age. Parity was not independently associated with physical performance (models 2 – 3). Sensitivity analyses with different cut-off values for parity, as well as assessing parity as a continuous variable, did not alter the results. The relation of early maternal age at first birth to poor physical performance was no longer significant (p = 0.07) once chronic diseases were added to the model. In model three, all chronic diseases except for osteoporosis and high blood pressure were significantly and independently associated with poor physical performance. In all models, there was a strong and significant association between the study sites Tirana and Natal and poor physical performance. The relationship became stronger after controlling for chronic diseases, despite the greater overall prevalence of chronic diseases in Latin America and Tirana compared to Kingston.

**Table 4:** Association between age at first birth and poor physical performance, defined as SPPB ≤8 (N = 907)

	**Model 1**		**Model 2**		**Model 3**	
	**OR (95% CI)**	**p-value**	**OR (95% CI)**	**p-value**	**OR (95% CI)**	**p-value**
Age 1st birth						
≤18	1.81 (1.21 – 2.69)		1.75 (1.17 – 2.64)		1.48 (0.96 – 2.28)	0.07
>18	1	<0.01	1	<0.01	1	
Age^1^	1.12 (1.06 – 1.18)	<0.01	1.12 (1.06 – 1.18)	<0.01	1.11 (1.04 – 1.17)	<0.01
Education						
≤8 years	1.59 (1.03 – 2.46)		1.58 (1.01 – 2.45)		1.72 (1.08 – 2.72)	
>9 years	1	0.04	1	0.04	1	0.02
Childhood economic adversity^2^	1.22 (1.02 – 1.46)	0.03	1.22 (1.02 – 1.47)	0.03	1.17 (0.97 – 1.42)	0.10
Study Site						
St Hyacinthe	1.08 (0.58 – 1.98)	0.82	1.07 (0.58 – 1.98)	0.82	1.05 (0.55 – 2.00)	0.88
Tirana	3.33 (1.89 – 5.87)	<0.01	3.33 (1.89 – 5.86)	<0.01	3.45 (1.86 – 6.40)	<0.01
Manizales	1.13 (0.58 – 2.21)	0.72	1.10 (0.56 – 2.17)	0.78	1.42 (0.70 – 2.89)	0.33
Natal	2.70 (1.41 – 5.18)	<0.01	2.69 (1.40 – 5.18)	<0.01	3.38 (1.66 – 6.87)	<0.01
Kingston	1		1		1	1
Parity						
≥3 children			1.04 (0.72 – 1.50)		1.06 (0.72 – 1.56)	
<3 children			1	0.82	1	0.77
Diabetes^3^						
Yes					1.68 (1.15 – 2.45)	
No					1	<0.01
Stroke						
Yes					2.76 (1.43 – 5.32)	
No					1	<0.01
Arthritis						
Yes					2.08 (1.47 – 2.97)	
No					1	<0.01
Cancer						
Yes					2.08 (1.10 – 3.93)	
No					1	0.02
Lung disease						
Yes					2.20 (1.41 – 3.45)	
No					1	<0.01
Coronary heart disease						
Yes					1.64 (1.11 – 2.41)	
No					1	<0.01
Osteoporosis						
Yes					0.98 (0.69 – 1.40)	
No					1	0.93
High blood pressure						
Yes					1.00 (0.70 – 1.44)	
No					1	0.99

^1^Age was centred at 64 because participants were only eligible for the study if they were between 64 and 75 years of age. The odds ratio corresponds to each addition year of age after 64.

^2^Childhood economic adversity scores can vary from 0 to 3. A point was given for each of the following self-reported childhood circumstances: poverty, hunger, and parental unemployment.

^3^Chronic diseases are self-reported measures.

In both models shown in Table 5, age at first birth is independently and significantly associated with the continuous SPPB score. Parity, categorized in any form, was not associated with the continuous SPPB score. The relation of maternal age at first birth to SPPB score was maintained after controlling for chronic diseases; having a child at 18 years of age or less was associated with a half point decrease in the total SPPB score. As above, diabetes, stroke, arthritis, cancer, chronic lung disease, and coronary heart disease were all significantly and negatively associated with the total SPPB score.

**Table 5:** Association between age at first birth and the continuous SBBP score, adjusted for age, parity, education, and childhood economic adversity (N = 907)

	**Model 1**		**Model 2**	
	**Estimate (95% CI)**	**p-value**	**Estimate (95% CI)**	**p-value**
Intercept	11.24 (10.79; 11.71)	<0.01	11.87 (11.39; 12.34)	<0.01
Age 1st birth				
≤18	-0.798 (-1.19; -0.37)		-0.52 (-0.92; -0.12)	
>18	-Ref-	<0.01	1	0.01
Study Site				
St Hyacinthe	-0.18 (-0.65; 0.30)	0.47	-0.17 (-0.62; 0.28)	0.47
Tirana	-1.51 (-2.00; -1.02)	<0.01	-1.38 (-1.87; -0.88)	<0.01
Manizales	-0.02 (-0.60; 0.57)	0.95	-0.20 (-0.76; 0.37)	0.49
Natal	-0.94 (-1.5; -0.35)	<0.01	-1.00 (-1.59; -0.41)	<0.01
Kingston	1	1	1	1
Diabetes^1^				
Yes			-0.56 (-0.90; -0.21)	
No			1	<0.01
Stroke				
Yes			-1.45 (-2.05; -0.85)	
No			1	<0.01
Arthritis				
Yes			-0.54 (-0.83; -0.25)	
No			1	<0.01
Cancer				
Yes			-0.46 (-1.00; 0.06)	
No			1	0.09
Lung disease				
Yes			-0.88 (-1.29; -0.46)	
No			1	<0.01
Coronary heart disease				
Yes			-0.56 (-0.92; -0.21)	
No			1	<0.01
Osteoporosis				
Yes			0.01 (-0.30; 0.32)	
No			1	0.96
High blood pressure				
Yes			-0.14 (-0.44; -0.17)	
No			1	0.38

^1^Chronic diseases are self-reported measure.

**Figure 2:**
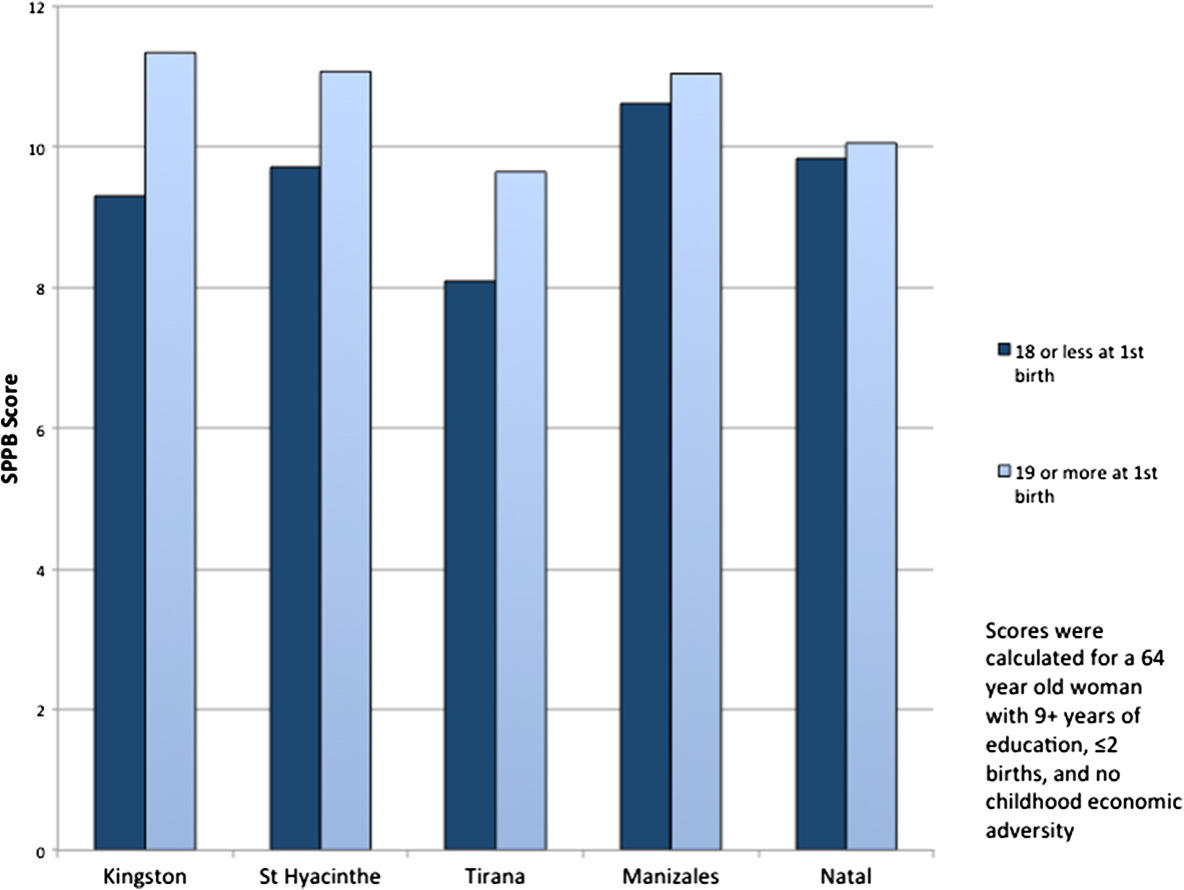
The effect of maternal age at first birth on physical function in older age, measured with the SPPB score, according to the study site.

There was effect interaction between maternal age at first birth and study site. Figure 2 presents the results of this model for a 64 year old women, with 9 or more years of education, 2 or fewer births and no childhood economic adversity. At the Canadian sites and in Albania, women who had their first child at 18 years of age or younger scored between 1.4 and 2.0 points lower on the SPPB scale than women who had their first child at 19 years of age or older. At the Latin American sites, there was little difference in SPPB scores between women who had their first child at a young age and women who had their first child at 19 or older. In fact, Canadian women who gave birth at 18 years or younger had lower SPPB scores than Latin American women in either reproductive exposure category.

## Discussion

In this study, we demonstrated that early maternal age at first birth was associated with poor physical performance in these women at older age, as well as an association between early maternal age at first birth and a number of chronic conditions: diabetes, chronic lung disease, and high blood pressure. Giving birth 3 or more times was associated with high blood pressure and chronic lung disease, but the relationship was not maintained after adjument for early maternal age at first birth. Thus, parity is likely on the causal pathway between age at first birth and these chronic conditions. We further demonstrated that part of the association between early maternal age at first birth and poor physical performance in older age was mediated by diabetes, chronic lung disease and possibly stroke. In the models using the continuous SPPB outcome, age at first birth was independently associated with SPPB, even after adjustment for chronic conditions. Women who gave birth at 18 years of age or younger scored, on average, half a point less on the SPPB than women who gave birth at 19 years of age or older. A half point less on the SPPB scale meaningfully reduces functional mobility [39]. Finally, we showed that the relationship between age at first birth and SPPB scores was heterogenous by study site. In contrast to our hypothesis, the effect of early maternal age at first birth on physical function was stronger at the Canadian and Albanian (>1 point difference on the SPPB) sites than the Latin American sites (<1 point difference). In a five-year prospective cohort study of elderly individuals, a single point decline on the SPPB scale was significantly associated with mortality [40].

Unlike parity [19,22,41-44], age at first birth is a relatively understudied potential determinant of chronic conditions in older age. One study in Belgium found a strong association between early maternal age at first birth and diabetes-related mortality (OR = 3.7) after controlling for age, education, and ethnicity [20]. Early maternal age at first birth has also been associated with cardiovascular risk factors, including high blood pressure. Of interest, this relationship has been seen in both men and women implicating a social, rather than biological pathway [45]. In our study, elevated parity seems to be a consequence of early maternal age at first birth. Previous studies documenting an association between parity and chronic conditions such as diabetes and cardiovascular disease [19,21,22,41] may be capturing the effect of early maternal age at first birth.

The effects of early maternal age at first birth on chronic diseases likely reflect both the biological and social consequences of bearing children as a teenager. Pregnancy and childbirth are marked by physiological adaptations that include insulin resistance, atherogenic dyslipidemia, fat accretion, and inflammation. Some of these manifestations may persist after giving birth [46]. For example, a common complication of childbirth- pre-eclampsia- is a known predictor of future cardiovascular disease [47] and young maternal age is well known risk factor for pre-eclampsia [48]. Additionally, women who begin childbearing early and have multiple children may accumulate excess weight over their lifetimes and experience chronic stress that contributes to cardiovascular and diabetes-related risk. The link between pregnancy and chronic lung disease has been less explored, although another study also found an association between early maternal age at first birth and lung disease [49]. With scant exception [50], almost no research has related reproductive history to physical function in older age. However, as posited in the introduction, obstetrical complications could directly affect mobility [24,51], while pregnancy-induced inflammation could increase the risk of mobility loss independent of chronic disease, as numerous inflammatory markers (CRP, IL-6, ILIRA) are associated with poor physical performance at older age [52].

Having a child at an early age may arise from and give rise to social consequences that increase the risk of developing chronic diseases and mobility loss in older age. For example, women who give birth as adolescents may not complete their educations because of childcare duties. Data from this study supports such a hypothesis. At all sites, women who gave birth at 18 years of age or less had lower educational attainment than women who gave birth at an older age. Compared to women who gave birth as adolescents, women who gave birth as adults had, on average, 4.6 years more education in Sainte Hyacinthe, 3.0 years more education in Tirana, 2.9 years more education in Kingston, 1.1 years more education in Manizales, and 1.0 more year of education in Natal. With less education, women have fewer employment opportunities and are at greater risk of poverty. Low educational attainment is also consistently associated with functional limitations [4,53-56]. As found elsewhere, the association between education and poor physical performance did not attenuate after controlling for chronic diseases suggesting a separate causal pathway possibly related to cumulative, lifetime socio-economic disadvantage [55].

In this study, heterogeneity across study sites in the association between early maternal age at first birth and physical performance was observed. Contrary to our hypothesis, the relationship was strongest in Canada and Albania. This finding may be attributable to survival bias at the Latin American sites. Most women in our study would have had their first child between 1955 and 1975 when maternal mortality was high in rural Latin American, but much lower in Albania and Canada. Childbirth at an early age is a known risk factor for maternal death [57], and linked to higher overall mortality later in life [58,59]. Many of these women may have died before this study began; complications of pregnancy and childbirth are the leading cause of death for women aged 15–19 in low and middle-income countries [57]. Furthermore, until recently, most of the population in Natal and Manizales had poor access to medical care. Women who had children as adolescents may have developed more chronic conditions and/or received substandard care for those conditions. Thus, in Latin America, mortality may have been disproportionately higher among women whose first birth was at an early age and who would have had poorer physical performance at an older age.

The unexpected results in Latin America may also reflect societal attitudes about teenage pregnancy. When women in this study would have started childbearing, teenage pregnancy was not unusual in Colombia and Brazil. In fact, it was and continues to be a way of increasing social class and visibility [60]. It is also a consequence of limited access to contraception [16]. In Canada during those same years, teenage marriage was unusual and most women who gave birth at 18 years or younger may have been unmarried. The social stigma accompanying teenage pregnancy would have been considerable, especially in Catholic Quebec [61]. Similarly, in Albania, childbirth outside of marriage was rare and would also likely have entailed significant social stigmatization [28]. We can expect that this stigma would have had lifelong consequences that manifested as chronic inflammation, stress response dysregulation and accelerated physical decline [62,63]. As a final consideration, populations in rural Brazil and Colombia have faced numerous other adversities across the life-course, including high levels of violence, which may have a greater influence on the development of physical limitations than does reproductive history [64,65].

There are a number of strengths to this study. IMIAS is comprised of community-based representative samples from 5 distinctive research sites in four countries, maximizing variability in life-course exposures. The sample size of 1040 women is large enough for most statistical procedures, allowing for relatively precise confidence intervals around estimates. Further, the SPPB is an objective outcome measure and has been extensively validated, including in Brazil and Quebec [33,37,38]. All instruments and questionnaires were pilot tested in Canada, Colombia, and Brazil in a preceding study.

There are limitations to this study. As mentioned above, there may be differential survival across study sites with the highest mortality expected from the Latin American sites. In Canada, the response rate was low (30%) since recruitment had to be done through contacts with family physicians to satisfy Canadian Ethics Review Boards. We thus compared educational attainment in our study samples from Canada with 2006 Canadian census data. Participants from Kingston had higher educational attainment than the reference population surveyed in the 2006 census, whereas differences were small in Saint-Hyacinthe. Given the consistently inverse association between educational attainment, chronic diseases, and poor physical performance, we may have under-sampled the group for which the association between early maternal age at first birth and poor physical performance is the most important. In other words, we may have underestimated the association in Kingston (Canada). Finally, exposure and covariate measures were self-reported. Among those with lower levels of education, it is possible that reports of provider-diagnosed chronic diseases were misunderstood or miscommunicated. To reduce misclassification, we described chronic diseases using both the medical and lay terms for each condition. We also assessed whether the associations between maternal age at first birth and high blood pressure, diabetes, and chronic lung disease were maintained in those with greater than 9 years of education compared to those with 8 or less years of education. Even when stratified by education, early maternal age at first birth was significantly association with each of these chronic diseases in both groups.

## Conclusions

This study provides evidence that women who give birth at a young age may be at greater risk of developing chronic diseases and physical limitations in older age. In our international study this was particularly true for women from Canada and Albania. These findings could improve the targeting of at-risk women by health professionals. If this relationship is causal, it reinforces the importance of providing contraception and sex education to young women, as some negative consequences of early pregnancy may be life-long.

## Abbreviations

IMIAS: International Mobility in Aging Study; SPPB: Short Physical Performance Battery.

## Competing interests

The authors declare that they have no competing interests.

## Authors’ contributions

MVZ and BA are principal investigators for the IMIAS study. CP and MVZ conceived of the present study and analysis plan. CP and ACPAS analyzed the data with feedback from all authors. CP also wrote the manuscript. All authors contributed to the interpretation of the data and to critical review of the manuscript.

## Pre-publication history

The pre-publication history for this paper can be accessed here:

http://www.biomedcentral.com/1471-2458/14/293/prepub
